# Advances in Cuffless Continuous Blood Pressure Monitoring Technology Based on PPG Signals

**DOI:** 10.1155/2022/8094351

**Published:** 2022-10-01

**Authors:** Caijie Qin, Xiaohua Wang, Guangjun Xu, Xibo Ma

**Affiliations:** ^1^Institute of Information Engineering, Sanming University, Sanming, China; ^2^CBSR&NLPR, Institute of Automation, Chinese Academy of Sciences, Beijing, China; ^3^Department of Nephrology, The Second Medical Center, Chinese PLA General Hospital, Beijing, China; ^4^Data Center, Agricultural Bank of China, Beijing 100049, China; ^5^School of Artificial Intelligence, University of Chinese Academy of Sciences, Beijing 100049, China

## Abstract

**Objective:**

To review the progress of research on photoplethysmography- (PPG-) based cuffless continuous blood pressure monitoring technologies and prospect the challenges that need to be addressed in the future.

**Methods:**

Using Web of Science and PubMed as search engines, the literature on cuffless continuous blood pressure studies using PPG signals in the recent five years were searched.

**Results:**

Based on the retrieved literature, this paper describes the available open datasets, commonly used signal preprocessing methods, and model evaluation criteria. Early researches employed multisite PPG signals to calculate pulse wave velocity or time and predicted blood pressure by a simple linear equation. Later, extensive researches were dedicated to mine the features of PPG signals related to blood pressure and regressed blood pressure by machine learning models. Most recently, many researches have emerged to experiment with complex deep learning models for blood pressure prediction with the raw PPG signal as input.

**Conclusion:**

This paper summarized the methods in the retrieved literature, provided insight into the artificial intelligence algorithms employed in the literature, and concluded with a discussion of the challenges and opportunities for the development of cuffless continuous blood pressure monitoring technologies.

## 1. Introduction

Cardiovascular diseases (CVD) have become the number one threat to human health, claiming 10.4 million lives worldwide each year [1]. Of all its causes, hypertension is the most dangerous one, because abnormal blood pressure (BP) can cause damage to vital organs such as the heart, brain, kidneys, and retina and even lead to myocardial infarction, cerebral hemorrhage, kidney failure, and other critical symptoms [2]. In recent years, with the aging population, the prevalence of obesity, and other factors, the prevalence of hypertension is on the rise around the world. According to the report of World Health Organization (WHO), the number of hypertension patients has doubled in the past 30 years and now exceeds 1.2 billion [3]. Moreover, some studies have shown that an increasing number of children and adolescents have abnormal blood pressure which causes negative effects on their health. Therefore, with its high morbidity, disability, and mortality rates, cardiovascular disease caused by blood pressure abnormalities has imposed a heavy burden on humans worldwide and became a major public health problem.

### 1.1. Introduction to Conventional Technologies

Prevention is the main way to control the prevalence of hypertension. Through the real-time detection of blood pressure and timely intervention of blood pressure, the risk of cardiovascular complications and death can be greatly reduced. Blood pressure measurement methods are divided into invasive measurement and noninvasive measurement methods. Therein, the former method implants a pressure sensor catheter into the aorta to detect pressure changes [4]. This method produces accurate results but is expensive, painful, and not suitable for routine measurement. At present, the commonly used noninvasive blood pressure measurement methods include the Korotkoff sound, oscillometric, arterial tension, and volume compensation methods.

1. Korotkoff sound method blocks arterial blood flow by inflating cuff and then uses a stethoscope to identify the percussive sound of blood flow reopening the vessel, while measuring the external pressure values detected by a manometer [5]. The Korotkoff sound method is the reference standard for cuff intermittent blood pressure measurements, but it is easily disturbed by external interference and relies on measuring physician's proficiency
2. The oscillometric method is similar to the Korotkoff sound method in that the arterial vessel is blocked by cuff inflation, and subsequent deflation of the cuff allows the vessel to flow again. Instead of detecting the sound, a pressure sensor on the air band detects the arterial pressure superimposed on the cuff [6]. The main disadvantages of oscillometric method are that it is sensitive to motion and needs the subject to remain stationary during measurement. Therefore, both the oscillometric method and the Korotkoff sound method need the subjects to wear a cuff, and thus, only intermittent single-point blood pressure measurements can be performed
3. Arterial tension method applies a certain pressure to the arterial vessel near the skin surface of the human body to cause a deformation of the vessel wall [7]. When the external pressure reaches a fixed value, the pressure inside the arterial vessel will be equal to the pressure on the skin surface, and the external pressure value can be considered the blood pressure. However, this method should ensure that the pressure transducer is placed directly above the artery and remains relatively stationary, which restricts its development
4. Volume compensation method detects vascular blood volume by a finger cuff with a photoelectric volume sensor [8]. With the constant change of external pressure acting on the blood vessel wall by the cuff, the regulating system makes the blood vessel blood volume constant. Therewith, the external pressure of the cuff is equal to the intravascular pressure, and blood pressure can be measured indirectly through the external pressure detection. However, this method needs to apply pressure to the measurement site of the human body, which may cause venous congestion during prolonged measurement, so it is not suitable for continuous measurement

Blood pressure is a dynamic physiological parameter, which has a diel rhythm [9] and is easy to fluctuate greatly with emotional changes or external stimuli [10]. A single intermittent blood pressure measurement usually cannot reflect the individual's physiological or pathological conditions. As such, continuous monitoring of blood pressure is of great significance. Otherwise, the cuff measurement methods usually cause discomfort to the user, so they are not suitable for infants, patients with open wounds (skin burns or ulcers, etc.), and people with large arm circumferences. Consequently, the research on cuffless continuous blood pressure monitoring is an urgent and significant task.

### 1.2. Photoplethysmography

Photoplethysmography (PPG) is a noninvasive method to detect the blood volume changes in tissues by photoelectric means. This method is based on the Lambert-Beer law [11], which indicates that the light passing through the blood tissue will be attenuated by the length of the propagation path, the density of the tissue, the absorbance, and other factors. The blood volume varies periodically with the blood ejection of the heart, as does the light intensity through the skin [12]. PPG waveform reflects abundant information about the cardiovascular function of the subject, and its formation is closely related to blood pressure as theoretically proven [13]. Moreover, since PPG signal can be easily collected with a photoelectric sensor, the prediction of blood pressure by PPG signal is an attractive and promising research direction.

**Figure 1:**
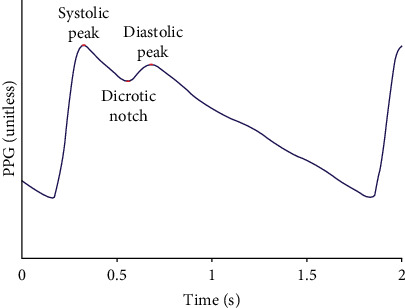
A common PPG pulse waveform.

As shown in Figure 1, the common PPG pulse waveform comprises an ascending branch and a descending branch. The ascending branch reflects the process of arterial expansion during ventricular ejection, while the descending branch reflects the vascular retraction during late ejection. As the ventricles diastole, the intraventricular pressure becomes lower than the blood pressure in the aorta, whereupon the blood flows back, followed by the closure of the aortic valve and a forward flow of blood. Therefrom, a smaller rise is formed in the descending branch, exhibiting a diastolic peak on the waveform.

PPG signals are cheap to acquire, easy to build into wearable devices (phones, smartwatches, etc.), and free of cuff limitations. Because of the urgent need for continuous cuffless blood pressure monitoring, PPG has attracted numerous attentions of the medical community all over the world, and a large number of researchers have conducted in-depth researches in recent years. Using Web of Science and PubMed as search engines, this paper reviewed PPG-based continuous cuffless blood pressure monitoring technologies over the past five years, focused on artificial intelligence-based solutions, and summarized specific implementations that could be used for reference.

## 2. Materials

### 2.1. Datasets

In the field of blood pressure prediction, researchers have released some open datasets that provide a data basis for fair comparison of different algorithms. Medical Information Mart for Intensive Care (MIMIC) is the most frequently used public dataset, which is a collaborative effort of physicians and computer science experts from Beth Israel Deaconess Medical Center (BIDMC), Massachusetts Institute of Technology (MIT), Oxford University, Massachusetts General Hospital (MGH), and others [14]. The MIMIC dataset provides physiological data such as electrocardiogram (ECG), photoplethysmography (PPG), arterial blood pressure (ABP), and respiration signals (RESP) collected from intensive care unit (ICU) wards. MIMIC III V1.4, as the most commonly used dataset version, contains data on 53,423 adult patients admitted to ICU between 2001 and 2012 and 7,870 neonates admitted between 2001 and 2008. All data in MIMIC are freely available to researchers worldwide and can be accessed through PhysioNet (http://physionet.org/).

The BP dataset in the University of California Irvine machine learning repository (UCI_BP) is derived from MIMIC II, and the data is stored in the v7.3 mat format of MATLAB [15]. This version of the dataset is also widely used because it has undergone some preprocessing and validation.

Some studies are based on the Queensland Vital Signs dataset [16], a monitoring dataset from 32 patients, all of whom used arterial blood pressure monitoring lasting from 13 minutes to 5 hours. The dataset also saves the corresponding data signal visualization results for easy viewing and use.

Another dataset used for blood pressure prediction is clinical data from patients admitted to Guilin People's Hospital in Guilin, China, and can be downloaded through the Figshare repository [17]. This dataset (PPG_BP, Figshare) contains statistics on 657 PPG segments of 219 subjects with a sampling frequency of 1000 Hz, which can be used for in-depth study of the relationship between PPG signals and blood pressure.

There are also some researches based on self-collected datasets, which explored the relationship between PPG signals and physiological parameters to some extent, but most of these datasets are small due to the acquisition device and cost.  Table 1 illustrates the datasets commonly used in the retrieved literature. As the collection time of each subject in the dataset is long, these long-time signals are usually segmented according to the needs of algorithm processing, and the resulting short duration sequence is called the sample herein.

### 2.2. Evaluation Metrics

Blood pressure prediction is a regression analysis and can be evaluated by mean error (ME), mean absolute error (MAE), standard deviation (STD), and root mean square error (RSME). The calculation criterion of ME is shown in formula (1), which represents the arithmetic mean of the error between the predicted value (${\overset{\wedge }{y}}_{i}$) and true blood pressure (*y*_*i*_). The calculation criteria of the MAE and STD are shown in formulas (2) and (3). The MAE is the average of the absolute values of the errors between the predicted value (${\overset{\wedge }{y}}_{i}$) and true blood pressure (*y*_*i*_). The calculation criterion of the RMSE as shown in formula (4) is obtained by calculating the square root of the mean of the square of the difference between the predicted value (${\overset{\wedge }{y}}_{i}$) and true blood pressure (*y*_*i*_).

(1) $$\begin{array}{c} ME=\frac{1}{n}\overset{n}{\underset{i=1}{\sum }}\overset{\wedge }{y_{i}}-y_{i}, \end{array}$$

(2) $$\begin{array}{c} MAE=\frac{1}{n}\overset{n}{\underset{i=1}{\sum }}\left|{\overset{\wedge }{y}}_{i}-y_{i}\right|, \end{array}$$

(3) $$\begin{array}{c} STD=\sqrt{\frac{1}{n-1}\overset{n}{\underset{i=1}{\sum }}\left(e_{i}-\bar{e_{i}}\right)},e_{i}={\overset{\wedge }{y}}_{i}-y_{i}, \end{array}$$

(4) $$\begin{array}{c} RMSE=\sqrt{\frac{1}{n}\overset{n}{\underset{i=1}{\sum }}{\left({\overset{\wedge }{y}}_{i}-y_{i}\right)}^{2}}. \end{array}$$

Compared with ME, the MAE indicator eliminates positive and negative offsets, and the STD reflects the dispersion of errors. Hence, MAE and STD are a pair of commonly used evaluation metrics in blood pressure estimation. The American Association for the Advancement of Medical Instrumentation (AAMI) stipulates that the MAE of blood pressure measuring devices should be less than 5 mmHg and the STD should be less than 8 mmHg. Alternatively, the grading criteria established by the British Hypertension Society (BHS) are shown in Table 2, which specifies the percentages of cumulative frequency with errors less than 3 thresholds, respectively, and achieving grade A or grade B is in line with the conditions for clinical use.

## 3. Methods

### 3.1. Preprocessing

Whether from public datasets or self-collected datasets, the PPG signals obtained are complex and easy to overlap with noise. Taking the frequently used MIMIC as an example, the subjects are patients in ICU, and their physical conditions have complex impacts on the PPG signals. Therefore, preprocessing the PPG signals to obtain high-quality signals is an important link affecting the accuracy of both subsequent feature analysis and artificial intelligence models.

1. Filtering: PPG signal is a very weak signal with a frequency range of 0-25 Hz and easy to be disturbed by external factors as well as its collection device during the acquisition process. The noises mainly include baseline drift caused by body motion and breathing and industrial frequency disturbance caused by electromagnetic wave radiation. These noises have a serious impact on the analysis of the PPG signal; therefore, filtering operations are required. Many researchers adopted Butterworth filter, which allows signals within a specific frequency range to pass and suppresses signals outside the given frequency range. For instance, Aguirre et al. [18] used a Butterworth filter with the cut-off frequency set to 0.5-8 Hz. Ghosh et al. [19] used a third-order Butterworth bandpass IIR filter with the cut-off frequency set to 1.5-20 Hz. Tiloca et al. [20] adopted a Butterworth FIR filter to limit the PPG signal to the frequency band range of [0.5-6] Hz. Likewise, Slapničar et al. [21] employed a fourth-order Butterworth filter with the cut-off frequency of [0.5-8] Hz to eliminate baseline drift and high-frequency noise. Furthermore in the research by Slapničar et al. [22], a fourth-order Butterworth bandpass filter with the cut-off frequency of 0.5-4 Hz was adopted. Other studies such as El-Hajj et al. [23] chose discrete wavelet decomposition to set the decomposition coefficients of the low-frequency and high-frequency components to 0 and performed soft threshold denoising on the remaining decomposition coefficients. In the study by Li et al. [24], the discrete Fourier transform was used to convert the temporal signal to the frequency domain and filter out the low-frequency components by setting the cut-off frequency. Besides, the Chebyshev filter with the cut-off frequency of 10 Hz was adopted for PPG signal filtering in the research by Hasanzadeh et al. [25]
2. Segmentation: the duration of the collected PPG signals is different. For instance, many PPG signals in the MIMIC dataset last up to tens of hours. Thus, PPG signals are usually divided into windows, referred to herein as segments. The length of the split windows can be set according to the needs of the subsequent AI algorithm, ranging from a single cycle to tens of seconds
3. Signal quality assessment: the PPG signals are still complex and variable after denoising; hence, thorough criteria need to be established to remove signals with poor quality, thereby reducing the negative impacts of anomalous signals on subsequent AI algorithms. This task is challenging, and no consistent criteria have been established yet, which often needs to be determined empirically or experimentally. First, the poor signals can be removed by some empirical values, such as limiting systolic blood pressure (SBP) and diastolic blood pressure (DBP) to 80-180 mmHg and 60-130 mmHg, respectively [26, 27], deleting the PPG signals with heart rate below 60 beats per minute [28], and rejecting outliers by using quartile [29] or 3-sigma criterion [30]. Moreover, considering the periodicity of PPG, some researches adopted autocorrelation between PPG periodic signals [31], dynamic time warping [32], or high-order statistics, such as skewness and kurtosis [33, 34], to further standardize the quality of PPG signals
4. Normalization: converting the amplitude of the PPG signal to the range of [0-1] can simplify and enhance the analysis process of the PPG signal and ensure that the extracted values are meaningful and fair to the subsequent characterization process. The normalization methods of these studies are relatively uniform, with some using the method in formula (5) to convert the amplitude of the PPG signal to 0-1 [35] and others normalizing the PPG signal to have a mean of 0 and a variance of 1 [21]:

(5) $$\begin{array}{c} x_{i}^{\prime }=\frac{x_{i}-\min\left(x\right)}{\max\left(x\right)-\min\left(x\right)}, \end{array}$$

where *x*_*i*_′ refers to the amplitude of the sample after conversion, *x*_*i*_ refers to the original amplitude of the sample, and min(*x*) and max(*x*) are the minimum and the maximum amplitude in the set of samples, respectively

### 3.2. Methods

A dataset consisting of a large number of PPG segments (here also called samples) belonging to different subjects is obtained after preprocessing. Next, the dataset is divided into training and test sets (deep learning models may include training, validation, and test sets) according to proportion or other means and are fed into models for subsequent study. Most researches divided the training and test sets randomly by sample, while only a few researches divided the training and test sets strictly by subjects. The latter method is more recommended. Two factors are considered in the case: primarily, PPG segments belonging to the same subject are correlated [32]; thus, dividing the dataset only by samples may bring out impure results due to the fact that there are segments of the same subject in both the training and test sets. Secondarily, considering that we need to deploy the algorithm on mobile phones or wearable devices in the future, the users to be tested must be the unseen subjects in the training set. For the above reasons, it is more robust to divide the training and test sets by subjects.

#### 3.2.1. Blood Pressure Estimation Method Based on Pulse Wave Velocity or Transit Time

The propagation speed of pulse wave is mainly influenced by the compliance of the arterial vessels. When the blood pressure is high, the arterial compliance becomes worse, and the propagation speed of pulse wave becomes faster. Conversely, when the blood pressure is low, then the artery compliance increases, and the transmission speed of the pulse wave slows down. The equation for the propagation velocity of waves in an elastic tube of an ideal type is

(6) $$\begin{array}{c} c_{0}=\sqrt{\frac{Eh}{\rho D}}. \end{array}$$

Here, *E* is the elastic modulus of the artery, *h* is the thickness of the artery wall, *D* is the inner diameter of the artery, and *P* is the blood density [36]. Pulse wave velocity (PWV) or pulse transit time (PTT) is an attractive index for blood pressure estimation. PTT can be considered the interval of pulse wave transmission time at different sites of arteries, which is inversely proportional to PWV. Besides, the pulse arrival time (PAT) has resemblance to PTT, which can be calculated by the time interval between the R peak of ECG and the peak of PPG. There are two common measurement methods for PTT/PAT. One is to synchronously measure the ECG and PPG signals. Alternatively, multiple sensors can be deployed at the proximal and distal ends, thereby calculating the time interval between the PPG signals at the difference sites of the human body.

Once the PTT/PAT/PWV is calculated, blood pressure can be estimated with a simple formula, which is a common method in early researches. Viunytskyi et al. [37] recorded both single-channel ECG and PPG signals through a self-designed device. The adopted dataset included a total of 30 records consisting of the records collected by the self-designed hardware and extracted from the MIMIC database. Blood pressure was estimated by a simple linear equation with an RMSE of 5.71 for SBP and 5.13 for DBP. Lazazzera et al. [38] collected PPG signals from the wrist and fingertips through two sensors placed on the back and the front of the smartwatch. The time intervals of the two sites PPG were fed into a linear model to estimate blood pressure. The model was validated on 44 subjects, and the results almost satisfied the standard of AAMI. Kim et al. [39] employed two sensors to collect PPG signals at different locations of the same finger and estimated blood pressure by a simple model with the time difference between the two sites PPG. The error rate was stable at about 5% in a small experimental cohort, and the error rate referred to the ratio of the prediction error to the true BP value. The signal acquirement by Byfield et al. [40] is similar to that designed by Kim et al., and the time interval of the two sites PPG was subjected to a Gaussian regression model for blood pressure prediction. Otherwise, Tabei et al. [41] synchronously acquired the PPG signals at the index of the left and right hand through the cameras of two mobile phones and sequentially calculated the PTT to estimate blood pressure.

Some other studies combined phonocardiogram (PCG) [42], impedance plethysmography (IPG) [43], and ballistocardiogram (BCG) [44] to obtain two sites signals, thereby estimating blood pressure by PTT/PAT/PWV. However, both ECG signals and multisite PPG signals require additional sensors. Hardware consumption increases the difficulty of deployment of wearable devices, and it is particularly hard to build into mobile phones because of the difficulty of hardware changes to widely used phones.  Table 3 summarizes BP estimation methods based on PTT/PAT/PWV presented in the paper.

#### 3.2.2. Blood Pressure Estimation Method Based on Pulse Wave Analysis (PWA)

**Figure 2:**
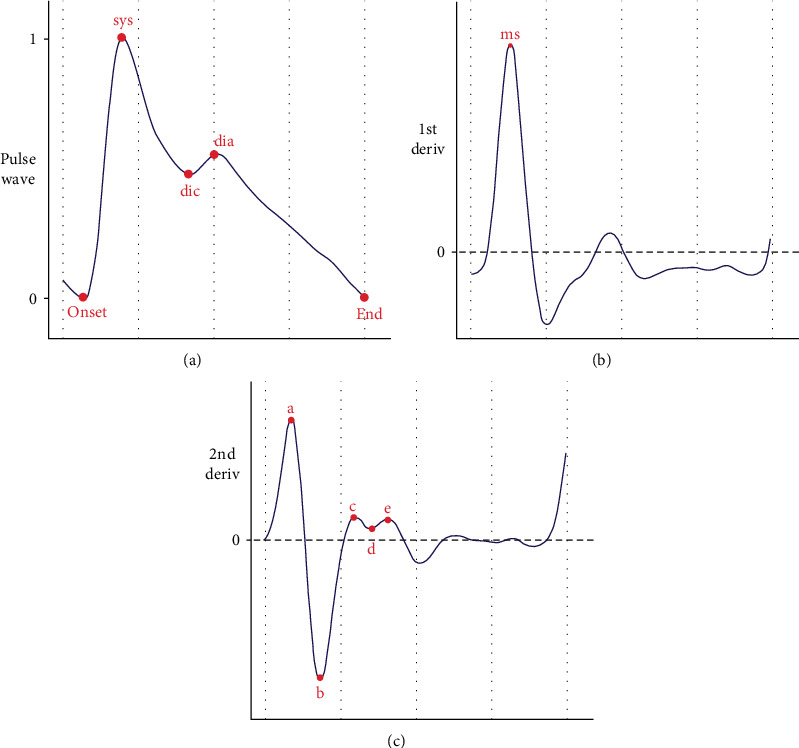
Fiducial points of PPG wave and its derivatives: The three subplots from top to bottom are (a) PPG pulse wave, (b) first-order derivative, and (c) second-order derivative. In (a), “onset” and “end” denote the beginning and the end of the waveform, respectively, “sys” and “dia” represent the systolic and diastolic peaks, and “dic” represents the dicrotic notch. In (b), “ms” denotes the maximum slope point. In (c), “a,” “b,” “c,” “d,” and “e” are five key fiducial points of the second derivative.

**Figure 3:**
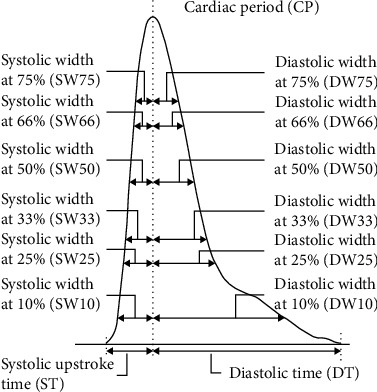
Pulse width feature of the PPG waveform.

The morphology of PPG waveform contains a wealth of physiological and pathological information and has a close relationship with the cardiovascular system. Pulse wave analysis (PWA) aims at expiring the physiological significance of the pulse wave by extracting rich features from PPG and its derivatives and combines with powerful artificial intelligence algorithms to estimate blood pressure. The paper [45, 46] provided prospective studies on the feasibility of using a single PPG signal to estimate blood pressure. Inspired by these researches, extensive studies are dedicated to mining BP-related features and combine traditional machine learning for blood pressure estimation. Researches of blood pressure estimation methods based on PWV focus on two aspects: One is mining features with strong correlation to blood pressure, and the other is the optimization of artificial intelligence algorithms. Many features of PPG waveform have been experimentally proven to be related to blood pressure, including the aforementioned PTT. The features adopted in the existing studies can be classified into time domain features, frequency domain features, demographic information, etc. As typical one among them, the peak of PPG waveform is considered related to stroke volume [47], while the pulse width at 50% and pulse area ratio are indicators related to total peripheral resistance [48]. In addition, the photoplethysmographic intensity ratio (PIR) can reflect variations in the internal diameter of the artery and correlates with changes in blood pressure [49], and the artery stiffness index (ASI) presents arterial stiffness [50]. Moreover, the *K* value can reflect the changes in peripheral resistance of blood vessels, elasticity of arterial walls, and blood viscosity [51]. The first derivative (VPG) and second derivative (APG) of PPG play an important role in detecting the fiducial points of PPG and analyzing the physiological significance of PPG waveform [52]. Therefore, abundant features can be extracted from the PPG derivatives for analysis. Furthermore, Fourier transform, singular value decomposition, wavelet transform, and other techniques can be adopted to map the time domain signal to the frequency domain, to perform spectral analysis of the PPG signal. Besides, entropy is a measure of signal uncertainty [53], so extracting the entropy of the PPG signal facilitates the analysis of complex blood pressure signals. Some of the typical features are listed in Table 4, and some of them are depicted in Figures 2 and 3.

In the existing studies, the maximum feature dimension extracted from PPG waveform reaches up to more than 200, among which redundant features will reduce the accuracy of the model and increase the complexity of the model. Hence, the feature selection method is advised to reduce the dimension of features, screen out the most relevant feature subset, and enhance the accuracy of the algorithm. Correlation analysis, multicollinearity analysis, recursive elimination, minimum redundancy maximum correlation, and intelligent optimization strategies such as genetic algorithm (GA) are all extensively used feature selection methods.

Machine learning algorithms can identify and analyze the complex mapping between PPG signals and blood pressure, thereby establishing an effective blood pressure prediction model. A large number of researches have applied different regression models to estimate blood pressure, such as multiple linear regression (MLR), regression tree (RT), random forest regression (RFR), support vector machine regression (SVR), Adaboosting regression, and artificial neural network (ANN). In particular, some researches have proved that random forest regression is a more robust algorithm because it can evaluate the importance of features and is not sensitive to outliers [54]. The BP estimation methods base on PWA retrieved in this paper are listed in Table 5.

Thambiraj et al. [55] extracted 43 features from ECG and PPG signals, employed a genetic algorithm to search for the optimal feature subset, and used a random forest model to estimate blood pressure. The MAEs of this method were 9.54 and 5.48 mmHg for SBP and DBP, respectively. Tiloca et al. [20] extracted 11 features including PTT, PIR, and heart rate from PPG and ECG signals in the MIMIC II dataset and employed a random forest regression model to predict blood pressure, which obtained a RMSE of 13.01 and 12.89 mmHg for SBP and DBP. The two aforementioned experimental datasets were not large and the experimental results did not meet the requirements of the BHS or AAMI standards. Hasanzadeh et al. [25] modified an algorithm for detecting the fiducial points of PPG signal, which helped to improve the accuracy of feature extraction. Based on 19 features such as heart rate, pulse width, and reflex index extracted from PPG signals, the blood pressure was estimated by linear regression, decision tree, random forest, and Adaboosting regression models. The validation results on the UCI dataset dedicated that Adaboosting and random forest regression outperformed other models. However, compared with the AAMI and BHS standards, the result of SBP could not satisfy the standards. Liu et al. [56] collected PPG and ECG signals from 35 clinical patients and extracted 15 relevant features to compare the performance of four regression models: decision tree, support vector machine, Adaboosting, and random forest. The results demonstrated that random forest regression outperformed the other models, with ME ± STD of SBP and DBP were 0.04 ± 6.11, and 0.11 ± 3.62 mmHg, respectively. Since the study only enrolled 35 subjects, the performance of the method needs to be verified on a large experimental cohort. Khalid et al. [57] extracted 5 features including area, time, and pulse width on PPG segments refined from the Queensland dataset, followed by a variance inflation factor (VIF) to perform a multicollinearity test on the features, and eliminated two redundant features. In this study, MLR, SVR, and RT were used to estimate blood pressure, and the results revealed that regression tree model achieved the best performance. A subsequent study by Khalid et al. [58] refined a comprehensive dataset of 18,010 PPG segments from Queensland and MIMIC datasets and extracted 16 time domain features from these PPG segments. The multicollinearity test was performed on the features by variance inflation factor (VIF), and the most significant 3 features were screened out. Subsequently, the *k*-nearest neighbor model was first adopted to cluster hypotension, hypertension, and normotensive and then combined with the regression tree algorithm to estimate blood pressure. The results obtained in this study were in line with AAMI standards. The two studies both used manual check to determine the quality of the signal, which needs to be improved in the future. Similarly, after *k* means clustering with three features extracted from ECG and PPG signals, Farki et al. [59] employed gradient boosting, random forest, and multilayer perceptron regression methods to regress the blood pressure for each cluster. The method was validated on the MIMIC dataset and yielded an MAE of 2.56 for SBP and 2.23 for DBP, but the details of the dataset were not clearly disclosed. Haddad et al. [60] extracted 27 features from PPG and its derivatives based on the 30-second PPG segments of 28 subjects in the MIMIC dataset. The study constructed the MLR model for blood pressure prediction and resulted in an error of 6.10 ± 8.01 mmHg for SBP and 4.65 ± .22 mmHg for DBP. Manamperi et al. [61] extracted 53 features from PPG and its derivatives and predicted blood pressure through a 6-layer ANN model. The experimental results on the MIMIC II dataset showed that both SBP and DBP achieved the grade A of the BHS standard. Subsequent trials with 50 voluntary subjects achieved the grade A of the BHS standard for DBP and grade B for SBP. Attarpour et al. [62] collected the wrist and fingertip PPG signals from 111 volunteers and then extracted a total of 34 features from PPG signal and its second derivative, height, weight, etc. Subsequently, the optimal feature subset was selected by using the moving backward algorithm and genetic algorithm, and finally, the blood pressure was predicted by a multilayer neural network. By comparison, a genetic algorithm-based feature selection algorithm improved the performance of blood pressure estimation, with an accuracy (MAE ± STD) of SBP as 5.59 ± 0.30 mmHg and DBP as 4.45 ± 0.16 mmHg. Chakraborty et al. [63] collected 670 records from 50 patients in the open dataset and extracted 15 time domain features. The study adopted neighborhood component analysis (NCA) and relief (RLF), respectively, to finally select the optimal feature subset consisting of four features. Based on the modified ANN model, the proposed algorithm obtained an error of 0.461 ± 2.62 mmHg for SBP and 0.15 ± 4.482 mmHg for DBP, whereas the first minute initial calibration and the four-minute blood pressure estimation made the method not very real-time, and the fiducial detection technique adopted in the study was sensitive to noise. Yang et al. [64] extracted 90 features including PAT, heart rate, and complexity features of PPG and ECG signals and employed support vector machine, Lasso, and artificial neural network models to predict blood pressure. This study validated on 14 male volunteers, and the optimal result (MAE ± STD) of SBP was 7.33 ± 9.53 mmHg obtained by the support vector machine model, while the optimal result of DBP was 5.15 ± 6.46 mmHg obtained by the ANN model. Otherwise, Chen et al. [65] extracted 14 features of ECG and PPG from the open dataset, selected features by the mean impact value (MIV) index, and finally predicted blood pressure using SVR model optimized by a genetic algorithm. The proposed algorithm yielded an error (MAE ± STD) of 3.27 ± 5.52 mmHg for SBP and 1.16 ± 1.97 mmHg for DBP. Similarly, Tan et al. [66] synchronously collected PPG and ECG from 10 healthy volunteers, extracted 17 time domain features, and retained features with the cumulative contribution rate more than 85% by MIV. The study used a GA-based BP network to model SBP and DBP separately, and the proposed algorithm outperformed the traditional regression model and ANN. Since the data size in the previous three studies was not big enough, the generalization ability and confidence of the model would be affected to some extent.

The following two researches dedicated efforts to explore the features related to blood pressure estimation. Chowdhury et al. [67] adopted the public dataset consisting of 657 PPG samples from 219 subjects and extracted 107 features including time domain, frequency domain, time-frequency domain, and demographic information. In this study, correlation evaluation, ReliefF, and minimum redundant maximum correlation were adopted for feature selection, and five machine learning methods, linear regression, regression tree, Gaussian process regression (GPR), support vector machine regression, and ensemble tree regression, were used for blood pressure estimation. The ReliefF method combined with GPR outperformed other algorithms, with a RMSE of 6.74 and 3.59 mmHg for SBP and DBP, respectively. Dey et al. [68] collected PPG signals from 206 volunteers using Samsung Galaxy S6 mobile phones and extracted 233 features in the time and frequency domain from PPG and its derivatives, which is the study with the highest number of feature dimensions in the retrieved literature. The study employed a Lasso regression model and categorical modeling based on demographic information, which improved the accuracy of blood pressure predictions. However, the performance of the model does not satisfy the AAMI standard.

In addition to traditional machine learning models, some studies have used more complex deep learning networks for blood pressure estimation after extracting the relevant features of PPG signals. El-Hajj et al. [69] extracted a total of 22 time domain features from the PPG signals and their derivatives, combined Pearson's correlation, random forest feature importance, recursive feature elimination, and sequential forward selection methods, to finally select seven consistently accepted features that have the greatest impact on blood pressure estimation. The validation results on the UCI dataset pointed out that LSTM and GRU outperformed other models, which were promising and both met the AAMI criteria. In a subsequent study, El-Hajj et al. [23] refined 942 subjects from the MIMIC II dataset and extracted 52 features from PPG and its derivatives. Combined with Pearson's correlation, mutual information, and recursive elimination method, the optimal feature subset consisting of 24 features were selected. The research designed a complex deep learning model that involved a bidirectional RNN layer, a series of conventional recurrent layers, and an attention layer, which resulted in an error (MAE ± STD) of 4.51 ± 7.81 mmHg for SBP and 2.6 ± 4.41 mmHg for DBP. Li et al. [24] extracted seven features (including PTT) from PPG and ECG signals, combined with a deep learning model for blood pressure estimation. The first layer of the deep learning model is a bidirectional long short-term memory layer, followed by a multilayer LSTM with a residual module. The validation results of this method on the MIMIC II dataset showed that SBP and DBP met grades B and A of BHS standard, respectively. Senturk et al. [70] extracted time domain and frequent domain features from signals in the MIMIC II dataset, combined with chaotic features such as the Shannon entropy, sample entropy, and fuzzy entropy and then compared the performance of three machine learning algorithms in blood pressure estimation. The results demonstrated that the nonlinear autoregressive with exogenous input neural network (NARX-NN) was superior to other algorithms, with an ME ± STD of 0.0224 ± 2.211 mmHg for SBP and 0.0417 ± 1.2193 mmHg for DBP. However, the size of the dataset used was not clearly disclosed in the study, so there might be barriers to comparing results across studies.

#### 3.2.3. Blood Pressure Estimation Method Based on Deep Learning with Raw PPG

The methods of blood pressure estimation based on PWA may incorporate irrelevant features or not fully mine the information contained in the PPG waveform, and their results highly depend on the accuracy of fiducial point detection of the PPG waveform. In recent years, with the rapid development of deep learning, many researches tend to use the raw PPG signals as input and utilize the advantages of deep learning in extracting complex high-dimensional features and advanced convolution computing capabilities for continuous blood pressure monitoring.

Blood pressure estimation methods based on deep learning network use the raw PPG waveform as input, and since the derivatives of PPG also contain features related to blood pressure [71], many studies take the PPG derivatives as input of the model. Researches on deep learning-based blood pressure monitoring methods mainly focus on the optimization of deep learning models and variants. One of the most classic models is the CNN-LSTM model, which is also the most widely used in literature. The hybrid model uses the convolutional neural network (CNN) layer to extract the complex features of the PPG signals and model them in time series with the help of the long short-term memory (LSTM) layer. For instance, in the research by Tazarv et al. [72], CNN was used as a feature extraction module, and LSTM was responsible for modeling over the time series. The CNN-LSTM model was validated on 20 randomly selected subjects from MIMIC II, resulting in an error of 3.70 ± 3.07 and 2.02 ± 1.76 mmHg for SBP and DBP, while the validation results on the Queensland dataset also reached the grade A of the BHS standard and complied with the AAMI standard. Similarly, Mou et al. [73] employed a CNN-LSTM model for blood pressure estimation and validated on three subjects of the MIMIC dataset. The results showed that, compared with the traditional models, the proposed model had a significant improvement in both training time and prediction accuracy. Otherwise, Esmaelpoor et al. [74] employed a CNN-LSTM two-stage model, with the CNN structure for extracting the features of PPG in the first stage and the LSTM structure for modeling the sequence signal in the second stage. Considering the correlation between SBP and DBP, the SBP prediction was applied to the next stage of DBP prediction, and vice versa. The model was tested on 200 subjects in the MIMIC II dataset and resulted in an error (MAE ± STD) of 3.97 ± 5.55 and 2.10 ± 2.84 mmHg for SBP and DBP, respectively, which complied with the AAMI standard and reached the grade A of the BHS standard. Nevertheless, the dataset was not strictly divided by subjects, which affected the confidence of the experimental results. Baker et al. [75] utilized a CNN-LSTM model that combined the feature extraction capability of the convolution layer and the temporal data modeling capability of the LSTM layer, with PPG and ECG signals from the open dataset as input, resulting in an MAE of 4.41 and 2.91 mmHg for SBP and DBP, respectively. Tanveer et al. [76] proposed an ANN-LSTM network which consisted of an ANN for extracting features and two stacked LSTM layers for modeling sequence signals. The proposed model was tested on 39 subjects in MIMIC I and achieved an MAE of 1.10 mmHg for SBP and 0.58 mmHg for DBP. Besides, the network proposed by Leitner et al. [31] adopted a gated recurrent unit (GRU) instead of the LSTM on the basis of the CNN-LSTM framework, because GRU has similar functions and slightly different parameters with LSTM. The proposed model was validated on long-term data of 100 subjects in the MIMIC dataset, combined with transfer learning-based calibration techniques, and resulted in an MAE of 3.52 and 2.20 mmHg for SBP and DBP, respectively.

Some other researches have introduced an attention mechanism to assign appropriate weights between different input channels or input vectors; therefore, the deep learning model can focus on more meaningful information and then improve the prediction accuracy. Qiu et al. [77] incorporated the squeeze and excitation block (SE module) in a 25-layer ResNet, which assigned weights to channel dimensions and improved channel attention. The hybrid model took PPG and ECG signals as input and predicted blood pressure on two datasets that included 1216 and 40 subjects from the MIMIC dataset, respectively. The prediction results of the model on the two datasets both achieved the grade A of the BHS standard and satisfied the AAMI standard. Chuang et al. [78] screened 11,000 PPG and ECG segments from 45 subjects in the MIMIC dataset and introduced an attention mechanism to the CNN-LSTM model to identify meaningful features. The results showed that combining the time and frequency domain signals of PPG and ECG could fully obtain the intrinsic characteristics of the signals, which resulted in an error (MAE ± STD) of 2.94 ± 4.65 mmHg for SBP and 2.02 ± 3.81 mmHg for DBP. The two aforementioned studies used a combination input of PPG and ECG to predict blood pressure, which necessarily increased hardware consumption. Aguirre et al. [18] proposed a recurrent neural network (RNN) encoder-decoder structure with an attention module and integrated demographic information such as age and gender to improve the prediction of mean ABP pulse. Under the condition that the training set and test set were strictly divided according to subjects, the MAE of SBP and DBP reached 6.57 ± 0.20 and 14.39 ± 0.42 mmHg, respectively.

The residual network (ResNet) [79] proposed by Microsoft Labs in 2015 can well solve the problems of gradient disappearance and gradient explosion brought by the deepening of layers through the residual structure. Schrumpf et al. [80] compared the performance of AlexNet, ResNet, LSTM, and the model proposed by Slapničar et al. [21]. With PPG signals and their derivatives as input, ResNet outperformed other models under the condition of strictly differentiated subjects in training and test sets, with an MAE of 16.4 and 8.5 mmHg for SBP and DBP, respectively. However, the associated SDs are not presented. Furthermore, MAEs of 16.4 and 8.5 mmHg are relatively large, meeting neither AAMI nor BHS standards. After transfer learning-based calibration, the prediction performance was significantly improved. Based on PPG and ECG signals of 40 subjects selected from the MIMIC dataset, Paviglianiti et al. [81] compared the performance of three deep learning models, ResNet, LSTM, and WaveNet. The results pointed out that ResNet combined with three LSTM layers achieved the best prediction performance, with an MAE of 4.118 and 2.228 mmHg for SBP and DBP, respectively.

The PPG signals are one-dimensional physiological signals, while some studies have transformed them into two-dimensional images, thereby performing transfer learning through a model pretrained on ImageNet. Wang et al. [82] converted one-dimensional PPG signals into images by using the visibility graph (VG) approach. This innovative approach preserved the time-frequency information in the PPG signals and allowed transform learning using CNN models pretrained on the large database ImageNet. The proposed idea was validated on 348 records from UCI_BP dataset, and the pretrained AlexNet model outperformed other models, leading to an MAE of 6.17 and 3.66 mmHg for SBP and DBP, respectively.

Other deep learning models and their variants include the following: Treebupachatsakul et al. [83] performed Fourier transform on PPG and ECG signals in open datasets and used the amplitude and phase of PPG and ECG signals as input to a context aggregation network (CAN). The network resulted in a RMSE of 7.1455 and 6.0862 mmHg for SBP and DBP, respectively. However, the associated MAEs or SDs are not presented. Sadrawi et al. [84] compared the performance of two deep convolutional autoencoders, LeNet-5 and U-Net, and employed a genetic algorithm to optimize the integration of encoders in the cross-validation process. The method was evaluated on 18 subjects in a single center and yielded an MAE of 2.54 mmHg for SBP and 1.48 mmHg for DBP. The network proposed by Brophy et al. [85] was based on the GAN framework, which mainly consists of a generator with two layers of LSTM and a discriminator with four layers of CNN. Notably, the model is different from previous models in its ability to generate continuous ABP based on the PPG signal, rather than directly producing two values of SBP and DBP. The proposed model was trained on the UCI dataset and tested on the Queensland dataset with long-time data selected from one subject, resulting in an ME of 2.95 ± 19.33 mmHg in mean arterial pressure. However, the population considered was only one subjects, too few to reduce the confidence of the results. Otherwise, Slapničar et al. [21] took PPG alongside its derivatives as input and proposed a complex network model (spectrotemporal ResNet) combining the residual module with a spectrotemporal block, which could fully extract the temporal and frequency information of the signals. The proposed model was validated on over 700 hours of PPG signals from 510 subjects in the MIMIC III dataset, resulting in an MAE of 9.43 mmHg for SBP and 6.88 mmHg for DBP.

Furthermore, some researches have also experimented on the CNN with large convolutional kernels, which may enable the model to obtain larger effective receptive fields. Panwar et al. [86] proposed a deep learning framework consisting of CNN, LSTM, and fully connected layer, and adopted filters of size 9 × 1 in the CNN layers. When evaluated on 1557 subjects from the open dataset, the MAE and STD of the proposed model were 2.30 and 0.196 mmHg for SBP, while 3.97 and 0.064 mmHg for DBP. The hybrid network structure proposed by Yen et al. [87] was composed of multiscale CNN, LSTM, and dense layers, in which the CNN structures adopted 9 × 1 and 25 × 1 kernel filters, respectively. The model was tested on 1551 subjects from the UCI_BP dataset, resulting in an MAE of 2.942 ± 5.076 and 1.747 ± 3.042 mmHg for SBP and DBP, respectively.

Table 6 illustrates the BP estimation methods based on deep learning model presented in this paper.

## 4. Future Research Directions

Continuous monitoring of blood pressure will be an urgent task in the future, therefore deploying the function on mobile phones or other portable wearable devices to realize daily BP monitoring will have a broad application prospect. From the early simple regression based on multiplexed signals, to PPG feature extraction combined with artificial intelligence algorithms, and then to deep learning models with raw PPG signal most recently, researches on continuous cuffless blood pressure estimation have made great progress over time. However, the current technology still cannot meet the standards of practical applications. The major challenges that need to be addressed urgently include the development of large-scale heterogeneous datasets, mining of strongly correlated feature sets, optimization of lightweight efficient models, researches on personalized modeling technology, and rPPG-based blood pressure estimation technology.

1. Most of the retrieved studies are based on open datasets. For example, the MIMIC dataset is collected from patients in the ICU, whose complex physical conditions have various effects on blood pressure. However, there are differences in blood pressure changes between diverse groups, such as young people and those with cardiovascular disease. Some researches were based on self-collected datasets, but the small amount of data led to low confidence in the validation results. Therefore, it is urgent to establish large-scale heterogeneous datasets to improve the adaptability of the model to different populations, verify the results of the algorithm, and promote the optimization of the algorithm
2. Due to the easy acquirement, low cost, and convenient deployment of PPG signals, PPG-based blood pressure estimation methods have gained a strong momentum in recent years, and many studies dedicated to explore the relationship between PPG features and the physiological significance of blood pressure. Considering the individual difference of PPG signals, there is no set of features directly related to blood pressure has been generally employed, so it is necessary to explore PPG features with explicit physiological significance in the future. Additionally, the existing offline training model should be deployed on mobile phones or other wearable devices, and the balance between the complexity and accuracy of the algorithm needs to be considered. Therefore, the development of lightweight and accurate models is also a very challenging task in the future
3. Because of the differences of blood pressure among individuals, even deep learning models trained on large-scale datasets cannot fully learn them. Thus, within the acceptable conditions for commercial deployment, the accuracy of the model can be improved through individual calibration. Haddad et al. [88] adopted a straightforward approach that calibrated estimation of the blood pressure with simple offsets from the same subject. Schrumpf et al. [80] adopted the idea of transfer learning to fine-tune the specific layer of the model with a small amount of template data of the target subject. These methods may mitigate the systematic error in blood pressure estimation, but the mapping relation between the input signal and the estimation of blood pressure still relies on the performance of AI model
4. Remote photoplethysmography (rPPG) can be used to extract the PPG signal by using a camera to capture the periodic signal of skin color caused by the cardiac cycle. As smartphones become common devices, rPPG technology can be easily deployed on mobile phones for home-style daily blood pressure monitoring. In recent years, some prospective researches [89–91] have made some progress. Affected by the influence of video quality, subjects' head movement, illumination, and other factors, rPPG-based blood pressure monitoring technology faces great challenges in front-end data collection and processing. However, as rPPG technology is simple, feasible, and easy to deploy, it is still an appealing direction in the future

## 5. Conclusion

This paper retrieved the progress of research in the past five years on the PPG signal-based cuffless continuous blood pressure prediction technology. In conclusion, PPG is a promising and appealing technology with great potential for application in cuffless continuous blood pressure monitoring. Although diverse BP estimation methods such as that based on PTT/PAT/PWV, PWA, and deep learning have emerged and achieved some results, to reach the standard of commercial application, the continuous blood pressure monitoring technology based on PPG needs in-depth researches in the following aspects: the construction of heterogeneous large datasets, feature mining and optimization of lightweight model, personalized calibration technology, and rPPG technology.

## Figures and Tables

**Table 1:** Summary of the database retrieved.

Authors	Dataset	Subjects	Samples	Signals
Slapničar et al. [21]	MIMIC III	510	—	PPG
Aguirre et al. [18]	MIMIC III	1131	6478	PPG
Leitner et al. [31]	MIMIC III	100	—	PPG
Baek et al. [92]	MIMIC II	942	1912	PPG, ECG
Schlesinger et al. [93]	MIMIC II	329	136459	PPG
Wang et al. [94]	MIMIC II	72	58795	PPG
Yen et al. [87]	UCI_BP	1551	—	PPG
Panwar et al. [86]	UCI_BP	1557	—	PPG
Khalid et al. [58]	Queensland, MIMIC II	—	8133, 9877	PPG
Han et al. [33]	PPG_BP Figshare	—	116	PPG

**Table 2:** The standards of BHS.

		Cumulative error percentage		
		5 mmHg	10 mmHg	15 mmHg
BHS	Grade A	60%	85%	95%
	Grade B	50%	75%	90%
	Grade C	40%	65%	85%

**Table 3:** Summary of BP estimation methods based on PTT/PAT/PWV.

Authors	Signals	Position of sensors	Subjects	Results (mmHg)	
				SBP	DBP
Viunytskyi et al. [37]	PPG, ECG	Finger, chest	30 records	RMSE: 5.71	RMSE: 5.13
Lazazzera et al. [38]	PPG, PPG	Wrist, finger	5 + 44 subjects	ME: -1.52 STD: 9.45	ME: 0.39 STD: 4.93
Kim et al. [39]	PPG, PPG	Finger, finger	21 subjects	Error rate ≈ 5%	
Byfield et al. [40]	PPG, PPG	Finger, finger	26 subjects	MAE: 2.117 STD: 0.257	MAE: 2.935 STD: 0.721
Tabei et al. [41]	PPG, PPG	Finger, finger	6 subjects	MAE: 2.07 STD: 2.06	MAE: 2.12 STD: 1.85
Marzorati et al. [42]	PPG, PCG	Finger, chest	20 subjects	MAE: 3.06	MAE: 1.83
Huynh et al. [43]	PPG, IPG	Finger, wrist	15 subjects	RMSE: 8.47 STD: 0.91	RMSE: 5.02 STD: 0.73
Yousefian et al. [44]	PCD, BCG	Wrist, wrist	22 subjects	MAE: 7.6	MAE: 5.1

**Table 4:** A list of typical feature extracted from the PPG and its derivatives.

Feature		Description
Time domain	Amplitude	Amplitude of the fiducial points in Figure 2 (e.g., systolic peak, diastolic peak, and dicrotic notch)
	Time	Time interval between the fiducial points in Figure 2 (e.g., systolic peak, diastolic peak, dicrotic notch, and onset)
	Width	Systolic width at 10%, 25%, 33%, 50%, 66%, and 75% and diastolic width at 10%, 25%, 33%, 50%, 66%, and 75%, as shown in Figure 3
	Area	Systolic area, diastolic area, and their ratios
	Derivatives	Ratio of amplitude of fiducial points on first-order derivative and second-order derivative in Figure 2

Frequency domain		Amplitude and frequency of the first, second, and third peaks of the frequency domain signal

Demographic information		Age, gender, height, weight, body mass index (BMI), etc.

Entropy		Shannon entropy, spectral entropy, approximate entropy, sample entropy, etc.

Statistical characteristics		Mean, standard deviation, skewness, kurtosis, etc.

Others		*K* value, PIR, ASI, etc.

**Table 5:** The summary of BP estimation methods based on PWA.

Authors	Dataset	Signals	Features	Feature selection	AI algorithm	Result (mmHg)	
						SBP	DBP
Haddad et al. [60]	MIMIC I 28 subjects	PPG	27	—	MLR	MAE: 6.10 STD: 8.01	MAE: 4.65 STD: 6.22
El-Hajj et al. [23]	MIMIC II 942 subjects	PPG	52	Pearson's coefficient, mutual information, recursive elimination	Deep learning recurrent model	MAE: 4.51 STD: 7.81	MAE: 2.6 STD: 4.41
Li et al. [24]	MIMIC 50 subjects	PPG, ECG	7	—	Deep LSTM	MAE: 6.726 STD: 14.505	MAE: 2.516 STD: 6.442
Farki et al. [59]	MIMIC II	PPG, ECG	3	—	*k* means+ (RFR, gradient boosting regression, multilayer perception)	MAE: 2.56	MAE: 2.23
Senturk et al. [70]	MIMIC II	PPG, ECG	19	—	RNN, NARX-NN, LSTM	ME: 0.0224 STD: 2.211	ME: 0.0417 STD: 1.2193
Thambiraj et al. [55]	UCI_BP 3801 records	PPG, ECG	43	GA	RFR	MAE: 9.54	MAE: 5.48
Tiloca et al. [20]	MIMIC II	PPG, ECG	11	—	RFR	RMSE: 13.01	RMSE: 12.89
Manamperi et al. [61]	MIMIC II, self-collected 50 subjects	PPG	53	—	ANN	MAE: 4.8	MAE: 2.5
Hasanzadeh et al. [25]	UCI_BP	PPG	19	—	LR, decision tree, RFR, Adaboosting	MAE: 8.22 STD: 10.38	MAE: 4.17 STD: 4.22
El-Hajj et al. [69]	MIMIC II	PPG	22	Pearson's correlation, random forest feature importance, RFE, sequential forward search	Feedforward neural networks, LSTM, GRU	MAE: 3.23 STD: 4.74	MAE: 1.59 STD: 1.77
Khalid et al. [58]	Queensland, MIMIC 18010segments	PPG	16	VIF	KNN+RT	ME: 0.07 STD: 7.1	ME: -0.08 STD: 6.0
Yang et al. [64]	Self-collected 14 subjects	PPG, ECG	90		SVR, Lasso, ANN	MAE: 7.33 STD: 9.53	MAE: 5.15 STD: 6.46
Attarpour et al. [62]	Self-collect 111 subjects	PPG	34	Moving backword algorithm, GA	Multilayer neural network	MAE: 5.59 STD: 0.30	MAE: 4.45 STD: 0.16
Liu et al. [56]	Self-collected 35 subjects	PPG, ECG	15	—	DTR, SVR, Adaboosting, RFR	ME: 0.04 STD: 6.11	ME: 0.11 STD: 3.62
Chakraborty et al. [63]	MIMIC II 50 subjects	PPG	15	NCA, RLF	Modified ANN	ME: 0.461 STD: 2.62	ME: 0.15 STD: 4.482
Chen et al. [65]	MIMIC III	PPG, ECG	14	MIV	GA-SVR	MAE: 3.27 STD: 5.52	MAE: 1.16 STD: 1.97
Chowdhury et al. [67]	Figshare_BP 219 subjects	PPG	107	Correlation, RLF, minimum redundancy maximum correlation	LR, RT, Gaussian process regression, SVR, integration tree regression	RSME: 6.74	RSME: 3.59
Khalid et al. [57]	Queensland 8133 segments	PPG	5	VIF	MLR, SVR, RT	ME: -0.1 STD: 6.5	ME: -0.6 STD: 5.2
Dey et al. [68]	Self-collected 206 subjects	PPG	233	—	Lasso	MAE: 6.9	MAE: 5
Tan et al. [66]	Self-collect 10 subjects	PPG, ECG	17	MIV	GA-BP	RMSE: 2.114	RMSE: 1.30

**Table 6:** The summary of BP estimation methods based on deep learning.

Authors	Dataset	Signals	AI algorithm	Result (mmHg)	
				SBP	DBP
Tazarv et al. [72]	MIMIC II 20 subjects	PPG	CNN-LSTM	MAE: 3.70 STD: 3.07	MAE: 2.02 STD: 1.76
Chuang et al. [78]	MIMIC 45 subjects	PPG, ECG	CNN-LSTM+self-attention	MAE: 2.94 STD: 4.65	MAE: 2.02 STD: 3.81
Treebupachatsakul et al. [83]	UCI 812 samples	PPG, ECG	CAN	RMSE: 7.1455	RMSE: 6.0862
Mou et al. [73]	MIMIC 3 subjects	PPG	CNN-LSTM	MAE: 4.42 for ABP	
Paviglianiti et al. [81]	MIMIC 40 subjects	PPG, ECG	ResNet, LSTM, WaveNet, ResNet+LSTM	MAE: 4.118	MAE: 2.228
Slapničar et al. [21]	MIMIC III 510 subjects	PPG, derivatives	Spectrotemporal ResNet	MAE: 9.43	MAE: 6.88
Brophy et al. [85]	UCI_BP Queensland 6 subjects	PPG	GAN	MAE: 2.95 STD: 19.33 for MAP	
Aguirre et al. [18]	MIMIC 1131 subjects	PPG	RNN encoder-decoder + attention	MAE: 6.57 STD: 0.20	MAE: 14.39 STD: 0.42
Wang et al. [82]	UCI_BP 348 records	Image transformed from PPG	Pretrained AlexNet, Inception-V3, VGG-19	MAE: 6.17	MAE: 3.66
Esmaelpoor et al. [74]	MIMICII 200 subjects	PPG	CNN-LSTM	MAE: 3.97 STD: 5.55	MAE: 2.10 STD: 2.84
Baker et al. [75]	MIMIC III 200000 segments	PPG, ECG	CNN-LSTM	MAE: 4.41 STD: 6.11	MAE: 2.91 STD: 4.23
Qiu et al. [77]	MIMIC 1216 subjects	PPG, ECG	ResNet + SE	MAE: 3.70	MAE: 2.81
Leitner et al. [31]	MIMIC 100 subjects	PPG	CNN-GRU	MAE: 3.52	MAE: 2.20
Schrumpf et al. [80]	MIMIC 3750 + 625 subjects	PPG	AlexNet, ResNet, LSTM, model of Slapničar et al.	MAE: 16.4	MAE: 8.5
Yen et al. [87]	UCI 1551 subjects	PPG	CNN-LSTM	MAE: 2.942 STD: 5.076	MAE: 1.747 STD: 3.042
Tanveer et al. [76]	MIMIC I 39 subjects	PPG, ECG	ANN-LSTM	MAE: 1.10	MAE: 0.58
Panwar et al. [86]	MIMIC II 1557 subjects	PPG	CNN-LSTM	MAE: 2.30 STD: 0.196	MAE: 3.97 STD: 0.064
Sadrawi et al. [84]	Self-collected 18 subjects	PPG	GA + Lenet5/U-net	MAE: 2.54	MAE: 1.48

## Data Availability

No data were used to support this study.
